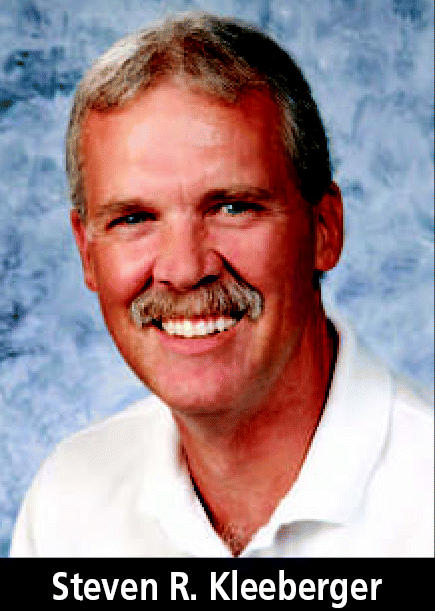# Interim Editors Bring Breadth of Experience

**Published:** 2007-03

**Authors:** Kenneth S. Korach

**Affiliations:** Interim Editor-in-Chief, *EHP*, E-mail: EHPeditor@niehs.nih.gov

As many of you are aware, Jim Burkhart retired as editor-in-chief of *EHP* in January of this year. Jim did an exceptional job of guiding the journal, and under his leadership *EHP* saw a rise in impact factor that now ranks the journal in first place in two categories of scientific publications: environmental sciences and public, environmental, and occupational health. Jim also believed strongly in the importance of *EHP* ’s news section and its use in international environmental health, and worked to maintain these efforts. I would like to take this opportunity to introduce myself, Matthew P. Longnecker, and Steven R. Kleeberger as the interim editor-in-chief and deputy editors, respectively, of *EHP*, and share with you our intentions for the journal during our editorial tenure, while a formal editor-in-chief is selected.

As some background, I currently serve as program director of the Environmental Diseases and Medicine Program, chief of the Laboratory of Reproductive and Developmental Toxicology, and chief of the Receptor Biology Section at the NIEHS. My research areas of interest include the basic mechanisms of estrogen hormone action in a variety of responsive tissues, with an application toward understanding how hormonally active environmental estrogens influence physiological processes; the role of the estrogen receptor in mediating hormonal responses; the estrogen receptor’s role in hormonal responsiveness during early development; the coupling of growth factor and nuclear receptor signaling pathways; and estrogen carcinogenesis and toxicity. I have been an editor and editor-in-chief of *Endocrinology*, the flagship journal of the American Endocrine Society.

Matt Longnecker is a board-certified internist with a doctorate in epidemiology who serves as a senior investigator in the Epidemiology Branch of the NIEHS. Matt’s early research focused on diet and cancer, especially the relationship of alcohol consumption to the risk of breast cancer. His current research program focuses on the health effects of early-life exposure to environmental agents. Matt serves on the editorial boards of the *American Journal of Epidemiology*, *Environmental Research*, and *Epidemiology*, and served for five years as an associate editor for the *Annals of Epidemiology*.

Steve Kleeberger serves as chief of the Laboratory of Respiratory Biology and director of the Environmental Genetics research group at the NIEHS. He is the principal investigator for the Director’s Challenge Program “Mechanisms of Susceptibility to Oxidative Stress–Induced Disease.” Steve’s research focuses on identifying genes that determine susceptibility to environmental lung disease. His work has led to the identification of significant susceptibility quantitative trait loci and functional characterization of candidate genes for susceptibility to lung injury induced by environmental pollutants. Steve’s laboratory is also focused on gene–environment interaction in human populations, and the pathogenesis of disease including coal workers’ pneumoconiosis, respiratory syncytial virus infection and chronic lung disease in infants, and acute respiratory distress syndrome in acute lung injury patients. Steve has served as a consultant to the World Health Organization and the Environmental Protection Agency regarding susceptible subpopulations and airborne pollutants. He has held a number of editorial positions, and is currently a reviewer for more than 20 peer-reviewed journals.

Through our various research endeavors, all of us have come to value *EHP* as an important voice and source for the field of environmental health science. During our tenure, journal operations will continue under *EHP* ’s established principles and policies, including particularly those addressing competing financial interests. We will continue to objectively and critically triage manuscripts, to work to reduce the time to first decision, and to provide a fair and objective review process for all manuscripts. We are investigating mechanisms for improving the manuscript submission and review process, and we will work to implement more effective reliance on *EHP* ’s editorial boards. Most importantly, we are dedicated to maintaining the high scientific quality and rigor of *EHP*, and the overall value of the journal and its programs to the environmental health science community.

## Figures and Tables

**Figure:**
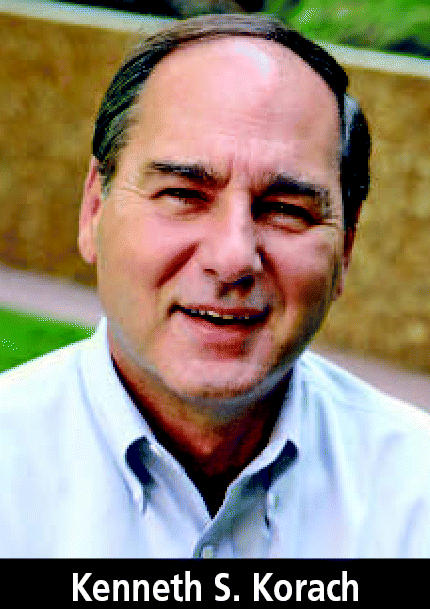


**Figure:**
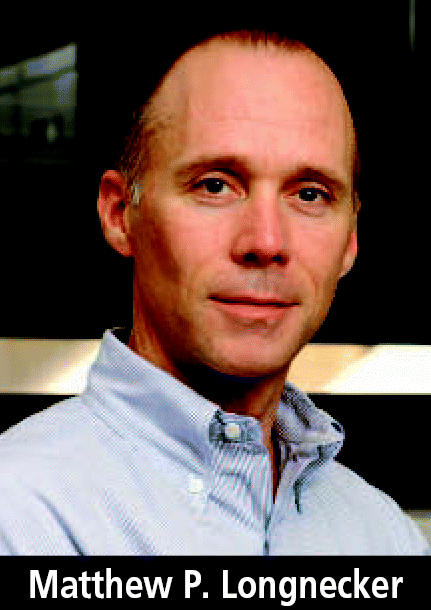


**Figure:**